# Maternal Sleep Disorders and Maternal and Birth Outcomes: A Retrospective US Claims‐Based Study

**DOI:** 10.1002/brb3.70908

**Published:** 2025-09-25

**Authors:** Anayeli Herrera Morales, Audrey C. Choh, Cici X. Bauer, Stefan A. Czerwinski, Miryoung Lee

**Affiliations:** ^1^ Department of Epidemiology, School of Public Health The University of Texas Health Science Center At Houston Brownsville Texas USA; ^2^ Department of Biostatistics and Data Science, School of Public Health The University of Texas Health Science Center At Houston Houston Texas USA; ^3^ School of Health & Rehabilitation Sciences, College of Medicine The Ohio State University Columbus Ohio USA

**Keywords:** birth outcomes | healthcare claims data | maternal outcomes | pregnancy | sleep disorders

## Abstract

**Introduction:**

Sleep disturbances are commonly reported during pregnancy and have been associated with adverse maternal, fetal, and neonatal outcomes. However, self‐reported sleep disturbances may not be accurately reflected in the prevalence of clinically diagnosed sleep disorders.

**Methods:**

Using Optum's de‐identified Clinformatics Data Mart Database, we conducted a cross‐sectional study of 93,767 American women who were pregnant with singletons between January 1, 2015 and June 30, 2021, to (1) determine the prevalence of clinically diagnosed sleep disorders and breathing abnormalities; (2) examine their associations with maternal health outcomes; and (3) examine their associations with birth outcomes. Sleep disorders and breathing abnormalities were defined on the basis of International Classification of Diseases (ICD)‐9 or ‐10 codes. Maternal and birth outcomes were defined on the basis of ICD‐10 codes. Multivariable binary and multinomial logistic regression models were used to examine associations, adjusting for demographic and insurance‐related factors, with additional adjustment for the infant's sex and pregnancy complications in birth outcome models.

**Results:**

The prevalence of clinically diagnosed sleep disorders and breathing abnormalities was 3.41%. These sleep conditions were significantly associated with increased odds (aORs: 1.25–3.37) of cesarean deliveries, gestational diabetes, gestational hypertension, preeclampsia, postpartum depression, stillbirths, newborn size by gestational age, birthweight, and gestation period among women with a singleton pregnancy.

**Conclusions:**

Our findings are consistent with previous research, but the lower prevalence of clinical diagnoses, compared to self‐reported rates, suggests underdiagnosis in clinical settings. This highlights the need for routine sleep screenings during prenatal care to support early detection and management. Key limitations include limited direct information on SES and restriction to an insured population. Future studies should explore these associations in more diverse and publicly insured populations to guide equitable screening and intervention strategies.

## Graphical abstract

In a retrospective US claims‐based study of 93,767 American women with singleton pregnancies, 3.41% were found to have clinically diagnosed sleep disorders. These sleep disorders were significantly associated with higher odds of adverse maternal, fetal, and neonatal outcomes compared to women without sleep disorders, highlighting the importance of sleep health in pregnancy.

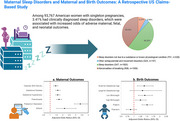

## 1 Introduction

Sleep disorders result from disturbances in the quantity, timing, and/or quality of sleep that continue to disrupt a normal sleep cycle and interfere with a person's daily functions (Pavlova Milena and Latreille 2019). Among the obstetric population, sleep disorders are often underdiagnosed because disturbances in sleep are considered common side effects of pregnancy. About 66%–97% of pregnant women report having some kind of sleep disturbance during their pregnancy as they experience many hormonal, physiologic, anatomical, and psychological changes that inevitably affect their sleep patterns (Coelho 2022; Driver and Sloan 2017; Balserak and Lee 2017).

Globally, the prevalence of sleep disturbances during pregnancy is high. A systematic review of epidemiological observational studies from 19 different countries found a pooled prevalence of 59.2% across pregnancy, increasing by trimester: 40.1%, 53.0%, and 83.9%, respectively (Moghadam et al. 2021). Similarly, studies from low‐ and middle‐income countries further emphasize the global burden of sleep disturbances in pregnancy. In India, a community‐based study showed nearly 50% prevalence of self‐reported sleep disturbances among pregnant women (Akashanand et al. 2025). In Ethiopia, 42% of pregnant women were found to have poor sleep quality, linked to socioeconomic and health‐related factors (Takelle et al. 2022). In contrast, the prevalence of clinically diagnosed sleep disorders is much lower. For instance, in the United States, the prevalence of self‐reported diagnosed sleep disorders among pregnant women was estimated to be lower compared to nonpregnant women, 3.9% versus 5.1%, whereas the overall prevalence among women of reproductive age (15–44 years) was estimated at 4.9% (Amyx et al. 2017). This stark contrast between self‐reported sleep disturbances and clinically diagnosed sleep disorders suggests substantial under‐recognition and under‐documentation in healthcare settings. As a result, many sleep disorders may go undiagnosed and untreated, missing critical windows for intervention.

The most prevalent sleep disturbances documented have been insomnia, sleep‐related breathing disorders, sleep‐related movement, and parasomnia, which could persist after childbirth (Moghadam et al. 2021). These disorders have been associated with short‐ and long‐term adverse health outcomes for both mothers and newborns, including hypertensive disorders, gestational diabetes, preterm birth, and low birthweight (LBW) (Steinweg et al. 2020; Wang et al. 2022; Yang et al. 2022; Lu et al. 2021; Li et al. 2018; Maghami et al. 2021; Brown et al. 2018; Warland et al. 2018; Maniaci et al. 2024; Komada et al. 2025). However, there are conflicting findings due to the heterogeneity in the definitions for the exposures and outcomes of interest, measurements, ascertainments, and the lack of adjustment for relevant confounders. These factors complicate comparisons and the integration of findings, highlighting the need for more standardized and large‐scale research approaches. Health insurance claims data offer a unique opportunity to address these limitations by offering comprehensive, large‐scale information on healthcare utilization, diagnoses, and outcomes across diverse populations in real‐world settings (Gavrielov‐Yusim and Friger 2014). Hence, through a secondary analysis of nationally representative healthcare insurance claims data and utilizing objective definitions based on the International Classification of Diseases Clinical Modification (ICD‐CM) codes, this study aimed to determine the prevalence of clinically diagnosed sleep disorders among pregnant women with singletons over a 6.5‐year period and to examine their associations with maternal and birth outcomes.

## 2 Methods

### 2.1 Data Source

We analyzed individual‐level healthcare data from Optum's de‐identified Clinformatics Data Mart Database, a compliant Health Insurance Portability and Accountability Act (HIPAA) closed system of administrative health claims in the United States. This large, adjudicated claims database includes data for inpatient and outpatient services as well as enrollment information for millions of individuals across all 50 US states, offering broad geographic and demographic representation of the insured population. The study team did not have full access to the database, and per our data use agreement, the extracted data for this study cannot be made publicly available. The study was reviewed by the Committee for the Protection of Human Subjects at the University of Texas Health Science School of Public Health, and it was determined exempt in accordance with the Common Rule (45 CFR 46.104(d)).

### 2.2 Study Population

In this secondary cross‐sectional analysis, we included women aged from 18 to 45 with singleton pregnancies that culminated between January 1, 2016 and June 30, 2020. To ensure data completeness, we included pregnant women who had continuous 1‐year enrollment before and after the culmination of their pregnancy. The 1‐year prepregnancy window was necessary to capture clinical diagnoses related to the exposures of interest (e.g., sleep disorders) and maternal conditions (e.g., gestational diabetes) associated with the pregnancy episode. The 1‐year post‐pregnancy window ensured adequate follow‐up for capturing postpartum depression and was also necessary to maintain the family identification number required to accurately link mothers and infants, allowing us to identify birth outcomes for the corresponding pregnancy. Therefore, the data available for eligible pregnant women ranged from January 1, 2015 to June 30, 2021, and for infants between January 1, 2016 and June 30, 2020.

### 2.3 Pregnancy Episode Identification and Verification

We applied the revised pregnancy identification algorithm developed by Ailes et al. (2023) to group pregnancy‐related claims into pregnancy records for each woman. The algorithm is based on a multistage process using the Ninth and Tenth revisions of the ICD‐CM and procedure coding systems (PCS) to identify pregnancy episodes, their gestational age (in weeks), and outcomes (e.g., live births, spontaneous abortions, induced abortions, and stillbirths) (Ailes et al. 2023). The service dates for the first and last pregnancy‐related claim records were used as a proxy to estimate each pregnancy episode's minimum and maximum dates. Additionally, the algorithm estimated the date of the woman's last menstrual period (LMP), the pregnancy delivery date, and the corresponding gestational age (in weeks). To group claim records into the same pregnancy episode, the algorithm implemented a gap requirement of a minimum of 120 days after any live birth record and at least 42 days after stillbirth and induced abortion records. After deduplication, the algorithm identified 97,936 pregnancy episodes for 96,793 women, resulting in 118 women being excluded from the pregnancy identification process.

**FIGURE 1:**
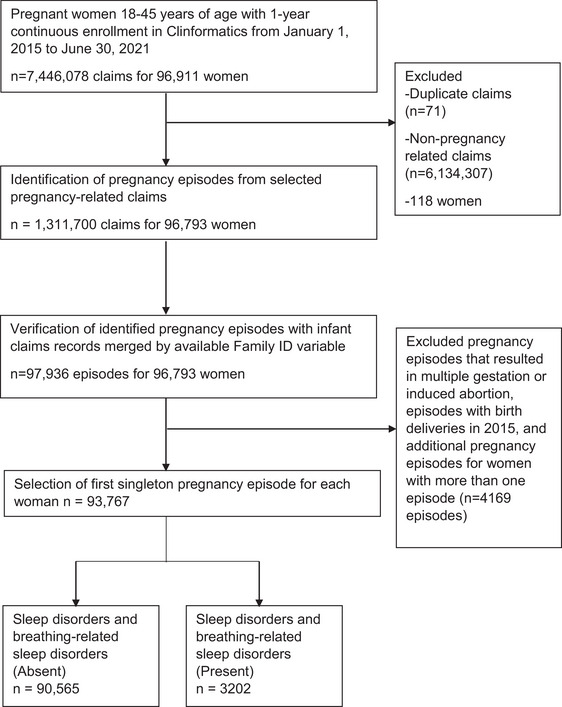
Flow chart depicting the selection process among pregnant women enrolled in Clinformatics from January 1, 2015 to June 30, 2021. The revised algorithm by Ailes et al. (2023) was applied to identify and verify the pregnancy episodes among pregnant women in Optum's de‐identified Clinformatics Data Mart Database during the study period.

We internally verified the identified pregnancy episodes by matching the mothers’ and infants’ records with the available family identification variable, which is an encrypted, system‐generated number that identifies a family unit. If a woman had more than one pregnancy episode during the study period, we applied a hierarchical process to prioritize singleton pregnancies with known outcomes: live births, stillbirths, spontaneous abortions, and unknown outcomes, in this order. We included unknown outcome pregnancy episodes as long as they did not have claim records with ICD‐10 codes O30 and O31, which pertain to multiple gestation pregnancies and their complications, respectively. We excluded pregnancy episodes that culminated in 2015 due to the availability of infants’ records starting in 2016 and those that resulted in induced abortions. We included only singleton pregnancies to reduce variability, as multiple gestations have higher risks and distinct impacts on sleep disorders (Santana et al. 2018; de la Calle et al. 2024). We excluded induced abortions because these pregnancies end before outcomes can be assessed, which is essential for our study's follow‐up objectives. After deduplication, our study sample size consisted of 93,767 pregnancies, corresponding to one eligible pregnancy episode per woman, as shown in Figure 1. To examine the association between clinically diagnosed sleep disorders and breathing abnormalities with maternal outcomes, we analyzed information for 93,645 pregnant women after excluding episodes with unknown pregnancy outcomes (*n* = 122). To examine the association between clinically diagnosed sleep disorders and breathing abnormalities and birth outcomes, we further excluded pregnancy episodes that resulted in stillbirths (*n* = 502) and those that could not be matched with the family identification number from the mothers’ records (*n* = 16,487), leaving 76,656 mother–infant dyads for data analysis.

**FIGURE 2:**
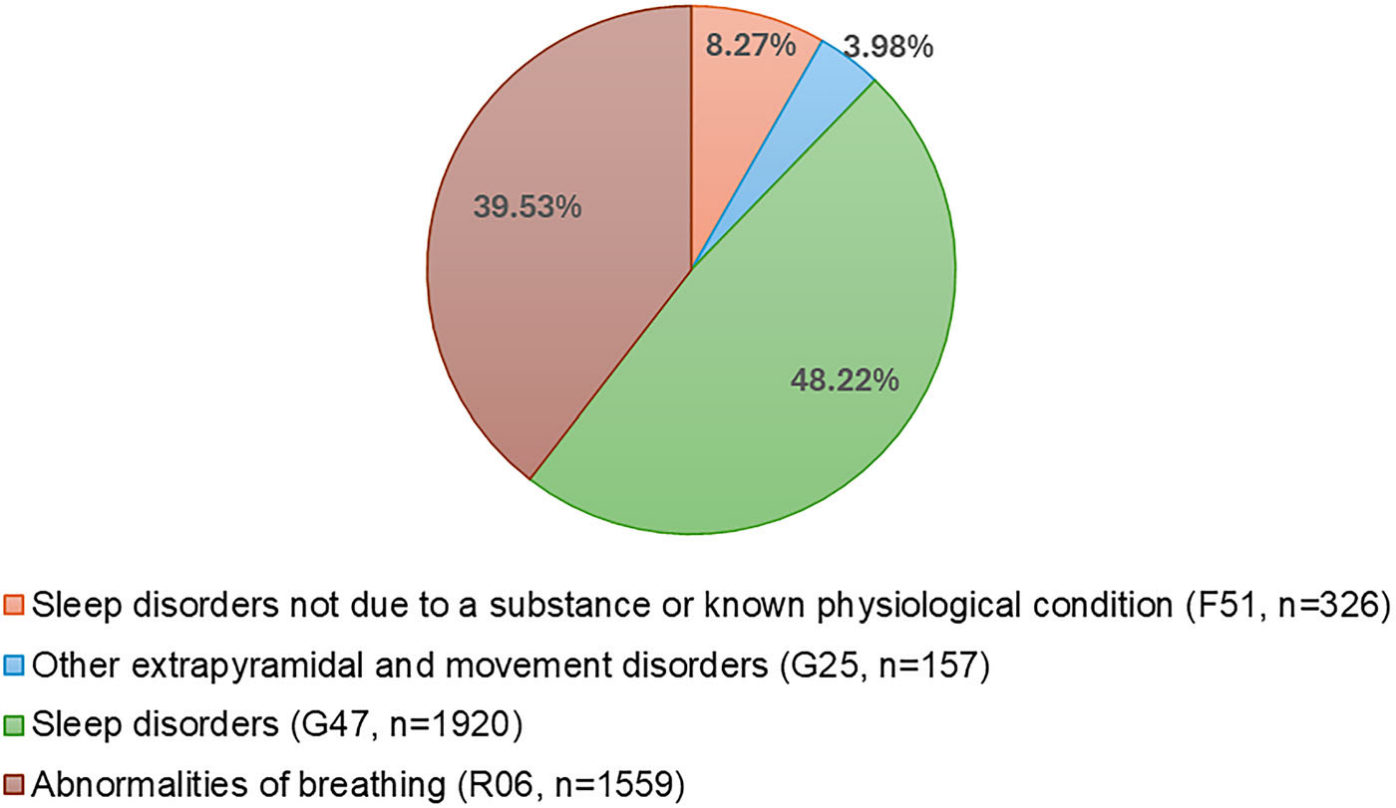
Distribution of sleep disorders and breathing abnormalities by ICD‐10 categories. The total number of identified diagnoses for sleep disorders and breathing abnormalities (*n* = 3944) exceeds the number of unique pregnant women (*n* = 3202) because some women were diagnosed with more than one type of disorder, categorized under different ICD‐10‐CM codes during the study period.

### 2.4 Study Measures and Variables

#### 2.4.1 Exposure: Sleep Disorders

Following ICD‐10‐CM categories, we identified sleep disorders and breathing abnormalities by extracting claim records that included F51 codes for sleep disorders not due to a substance or known physiological condition, G25 for other extrapyramidal and movement disorders, specifically G25.81 for restless leg syndrome, G47 codes for sleep disorders, and R06 codes for breathing abnormalities that could possibly lead to partial or complete airway obstruction due to the body position while sleeping. To capture sleep diagnoses from 2015, we converted ICD‐9‐CM codes (e.g., 307.4X, 327.23, 333.94, 780.5X, and 786.0X) to ICD‐10‐CM equivalents (American Medical Association 2008; World Health Organization 2016; The National Center for Health Statistics n.d.). Code selection was based on clinical relevance and availability in the claims data. Pregnant women were considered “exposed” if they had at least one claim with any of the above codes (Table S1) during the study period. This approach aligns with prior literature using administrative data and reflects the clinical reality that a single coded diagnosis typically indicates a provider‐identified condition relevant enough to be documented (Jolley et al. 2018). Given that sleep disorders in pregnancy are frequently underdiagnosed or overlooked, requiring multiple claims may have led to the underestimation of prevalence. Women without any relevant codes were considered “unexposed.” To avoid overestimating the prevalence of a specific condition, we counted one diagnosis per person, regardless of the number of claims each person had for that particular ICD‐10 code over the study period.

#### 2.4.2 Maternal Outcomes

We relied on ICD‐10‐CM codes to create binary variables (yes vs. no) for the presence of each maternal outcome of interest. We extracted ICD‐10‐CM codes for cesarean birth deliveries (e.g., O82, O75.82, and Z38.01), gestational diabetes (e.g., O24.4X), gestational hypertension (e.g., O13.XX), preeclampsia (e.g., O14.XX), and postpartum depression (e.g., F53.0). Stillbirths were identified using ICD‐10‐CM codes Z37.1 and P95 through a pregnancy identification and verification algorithm. To ensure that outcomes were attributable to the selected pregnancy episodes, we defined an outcome ascertainment period using the estimated start and end dates of each pregnancy. Postpartum depression was captured within 12 months following delivery, and an additional 6‐week window was included to identify postpartum preeclampsia (e.g., O14.15) (Putnick et al. 2020; “Treatment and Management of Mental Health Conditions During Pregnancy and Postpartum: ACOG Clinical Practice Guideline No. 5” 2023; Hauspurg and Jeyabalan 2022).

#### 2.4.3 Birth Outcomes

The three outcomes of interest were the size of the newborn by gestational age, birthweight, and gestation period based on ICD‐10‐CM codes from the infants’ records. We determined newborn size by gestational age by extracting ICD‐10 codes for small‐for‐gestational‐age (SGA) (e.g., P05.1X) and large‐for‐gestational‐age (LGA) (e.g., P08.0). According to ICD‐10‐CM, SGA refers to newborns whose weight and length are below the 10th percentile for gestational age, whereas LGA refers to “exceptionally large” newborns weighing more than 4500 g. Infants without these ICD‐10 codes were classified as appropriate for gestational age (AGA). If an infant had records indicating both SGA and LGA, the value for gestational age was marked as missing (*n* = 2). Similarly, we determined birthweight categories by extracting ICD‐10 codes for LBW (e.g., P07.0X and P07.1X) and high birthweight (HBW) (e.g., P08.1). LBW refers to newborns weighing less than 2500 g, whereas HBW refers to newborns weighing between 4000 and 4499 g. Infants without these ICD‐10 code were classified as having a normal birthweight. If an infant had records indicating both LBW and HBW, the value for birthweight was marked as missing (*n* = 36). Lastly, we determined infants’ gestation periods by extracting ICD‐10 codes for newborns with a gestation period of 28–37 completed weeks (e.g., P073.X), which were classified as preterm births, and newborns with a gestation period over 40–42 completed weeks (e.g., P08.21), which were classified as post‐term births. Infants without either ICD‐10 codes were classified as having a gestation period of 37–39 completed weeks. If an infant had records for both categories, the value for the gestation period was marked as missing (*n* = 17).

#### 2.4.4 Covariates

We additionally extracted demographic data from the available enrollment information, including the enrollee's birth year, race and ethnicity, state of residence, family identification number, and insurance‐related factors. The latter included information on the type of health plans and the financial arrangement for benefit plans and medical expenses. Administrative services only (ASO) enrollment (yes vs. no) indicated that the employer self‐funded the health insurance plans instead of purchasing a traditional insurance policy from an insurance carrier (The Association of Health Care Journalists n.d.). Consumer‐driven health plans (CDHPs) captured enrollment in high‐deductible health plans (HDHPs) with a tax‐advantaged account, such as Health Reimbursement Arrangements (HRAs) or Health Savings Accounts (HSAs), to cover medical expenses (The Association of Health Care Journalists n.d.). Health exchange (yes vs. no) indicated enrollment in public health insurance exchanges created by the Affordable Care Act in 2010 (The Centers for Medicare and Medicaid Services n.d.). Product type reflected the enrollee's selection among the different types of health insurance plans, such as exclusive provider organization (EPO), health maintenance organization (HMO), point of service (POS), preferred provider organization (PPO), or plans falling into the “Other” category. These four insurance‐related factors provided information on healthcare access and utilization that served as proxies for socioeconomic status due to the lack of detailed education, employment, or income level information (Armstrong et al. 2017; Lee et al. 2021).

Maternal age was calculated by subtracting the pregnant woman's birth year from the year derived from the pregnancy identification algorithm‐estimated delivery date and categorized into three age groups: 18–25, 26–34, and 35–45. These categories reflect commonly used groupings in obstetric research, where 26–34 is considered the reference range for lowest maternal risk, 18–25 captures younger maternal age often linked to social or developmental vulnerability, and 35–45 represents advanced maternal age, associated with increased risks for pregnancy complications (American College of Obstetricians Gynecologists' Committee on Clinical Consensus‐Obstetrics et al. 2022; Osterman et al. 2021; Laopaiboon et al. 2014). Gestational age for the studies was derived from the pregnancy identification algorithm that used ICD‐9‐CM 765.2X codes and ICD‐10‐CM Z3A.XX codes for specific weeks of gestation, which we categorized into three groups based on ICD‐10 categories: ≤36, 37–40, and 41–42 weeks. Geographic divisions were derived by categorizing states according to the predetermined groupings by the US Census Bureau (Bureau of the Census, US Department of Commerce n.d.).

#### 2.4.5 Statistical Analysis

We conducted Student *t*‐tests to compare continuous data, presented as means with corresponding standard deviations, and Chi‐squared tests for categorical data, tabulated as frequencies and percentages. As diagnoses for sleep disorders are not necessarily mutually exclusive, some women were diagnosed with multiple sleep disorders simultaneously during the study period. Therefore, to calculate the period prevalence of diagnosed sleep disorders, we performed an overall frequency count of unique pregnant women with any diagnosed sleep disorder and divided it by the total number of eligible pregnant women included in the data analysis. Maternal sleep disorders were analyzed as a dichotomous variable (yes vs. no) or by the number of sleep disturbances (zero, one, and two or more). Results are presented as odds ratios (ORs) with corresponding 95% confidence intervals (CIs). We performed multivariable logistic regression models to assess associations between maternal sleep disorders and maternal outcomes, adjusting for demographic and health insurance–related factors. For birth outcomes, multivariable multinomial logistic regression models were used, with additional adjustment for the infant's sex and pregnancy complications due to their known association with our birth outcomes of interest. Reference groups for birth outcomes were mothers of infants who were AGA, had normal birthweight, and were delivered at term. All statistical analyses were conducted using Statistical Analysis Software version 9.4 (SAS Institute Inc. Cary, NC) (SAS Institute Inc. 2023). A *p* value <0.05 was considered statistically significant.

## 3 Results

The complete demographic data for our study sample are provided in Table 1. Most pregnant women in the analysis were White (52.35%), between the ages of 26 and 34 (60.68%), from the South Atlantic (22.72%) and West South Central (21.21%) regions, delivered a term singleton (85.61%), and were enrolled in a fully insured healthcare plan (i.e., not in ASO plans, 69.90%), chose a CDHP without HRAs or HSAs (71.95%), received their insurance coverage through employers or other private means (i.e., not through the health exchange marketplace, 92.20%), and selected POS as their managed health insurance plan (70.25%). At a significance level of 0.05, pregnant women with (i.e., exposed to) and without (i.e., unexposed to) sleep disorders and breathing abnormalities differed in all demographic characteristics, except being enrolled in a fully insured healthcare plan and not having HRAs or HSAs. These demographic differences remained among the exposed (*n* = 3199) and unexposed (*n* = 90,446) pregnant women included in the data analysis for the association with adverse maternal outcomes. However, the exposed (*n* = 2574) and unexposed (*n* = 74,082) pregnant women included in the data analysis for the association with birth outcomes differed by all demographic characteristics, except geographic division and CDHPs.

**TABLE 1:** Demographic characteristics by sleep disorders status of pregnant women enrolled in Clinformatics between January 1, 2015 and June 30, 2021.

Variables	Total *N*	Sleep disorders (absent)	Sleep disorders (present)	*p* value
Overall mean (SD) or *n* (%)	93,767	90,565	3202	
Age at delivery (years)	30.12 (5.09)	30.09 (5.08)	31.04 (5.37)	<0.0001
Gestational age (weeks)^a^	38.40 (2.14)	38.42 (2.12)	37.97 (2.60)	<0.0001
Age group				<0.0001
18–25	17,712	17,211 (19.00)	501 (15.65)	
26–34	56,895	55,084 (60.82)	1811 (56.56)	
35–45	19,160	18,270 (20.17)	890 (27.80)	
Gestational age^a^				<0.0001
≤36 weeks	7778	7355 (8.14)	423 (13.24)	
37–40 weeks	80,274	77,623 (85.92)	2651 (82.92)	
41–42 weeks	5489	5367 (5.94)	122 (3.82)	
Race and ethnicity^b^				<0.0001
Asian	6204	6048 (8.01)	156 (5.60)	
Black	9754	9356 (12.39)	398 (14.30)	
Hispanic	13,250	12,836 (17.00)	414 (14.87)	
White	49,098	47,282 (62.61)	1816 (65.23)	
Geographic division^c^				0.0287
New England	1846	1771 (1.96)	75 (2.35)	
Middle Atlantic	5360	5198 (5.76)	162 (5.07)	
East North Central	12,507	12,072 (13.38)	435 (13.61)	
West North Central	8916	8624 (9.56)	292 (9.14)	
South Atlantic	21,301	20,541 (22.77)	760 (23.78)	
East South Central	4806	4618 (5.12)	188 (5.88)	
West South Central	19,890	19,259 (21.35)	631 (19.74)	
Mountain	10,140	9767 (10.82)	373 (11.67)	
Pacific	8657	8377 (9.28)	280 (8.76)	
Administrative services only^d^				0.0952
Yes	28,951	27,919 (30.85)	1032 (32.24)	
No	64,740	62,571 (69.15)	2169 (67.76)	
Consumer‐driven health plan^e^				0.3243
HRA	2973	2859 (3.25)	114 (3.69)	
HSA	20,726	20,041 (22.75)	685 (22.18)	
No HRA/HSA	67,463	65,174 (74.00)	2289 (74.13)	
Health exchange^f^				0.0007
Yes	7237	7040 (7.78)	197 (6.15)	
No	86,454	83,450 (92.22)	3004 (93.85)	
Product^g^				0.0174
EPO	12,018	11,614 (12.83)	404 (12.62)	
HMO	14,408	13,972 (15.44)	436 (13.62)	
Other	570	542 (0.60)	28 (0.87)	
POS	65,873	63,568 (70.25)	2305 (72.01)	
PPO	822	794 (0.88)	28 (0.87)	

Abbreviations: EPO, exclusive provider organization; HSA, health savings accounts; HMO, health maintenance organization; HRA, health reimbursement arrangement; POS, point of service; PPO, preferred provider organization.

^a^This variable was computed using the algorithm by Ailes et al. (2023) and categorized based on ICD‐10 codes. Missing (*n* = 226).

^b^Missing (*n* = 15,461).

^c^Missing (*n* = 344).

^d^Missing (*n* = 76).

^e^Missing (*n* = 2605).

^f^Missing (*n* = 76).

^g^Missing (*n* = 76).

We identified a total of 6570 claims for sleep and breathing abnormality disorders from January 1, 2015 to June 30, 2021. Following the one‐diagnosis‐per‐person approach, these claims corresponded to 3202 unique women, as shown in Table 2. The period prevalence of any clinically diagnosed sleep disorders and breathing abnormalities among pregnant women with singletons was 3.41%. The number of clinical diagnoses ranged from one to eight per woman. The majority of the exposed pregnant women (84.45%) had claims for one diagnosed disorder, and 15.55% of them had claims for two or more diagnoses. Because of this, the total number of diagnoses (*n* = 3944) exceeded the number of unique exposed pregnant women. By ICD‐10‐CM categories, 48.22% (*n* = 1902) of the diagnoses belonged to G47 codes for sleep disorders, 39.53% (*n* = 1559) to R06 codes for breathing abnormalities, 8.27% to F51 codes for sleep disorders that were not due to a substance or known physiological condition, and only 3.98% (*n* = 157) to G25 for other extrapyramidal and movement disorders, specifically for restless leg syndrome, as shown in Figure 2. The most common disorders were insomnia (*n* = 1222), dyspnea (*n* = 1377), and sleep apnea (*n* = 553). Table S1 presents the distribution for the 53 ICD‐10‐CM subcategories found in our study sample.

**TABLE 2:** Distribution of the number of sleep disorders among pregnant women enrolled in Clinformatics from January 1, 2015 to June 30, 2021.

Number of diagnoses	*n*	(%)
Any	3202	100.00
1	2704	84.45
2	356	11.12
3	84	2.62
4	30	0.94
5	18	0.56
6	5	0.16
7–8^a^	5	0.16

^a^In accordance with our data use agreement, we do not report exact diagnosis counts for cells containing fewer than five individuals.

Table 3 presents the unadjusted associations between clinically diagnosed sleep disorders and breathing abnormalities with adverse maternal outcomes. Pregnant women with sleep disorders had higher percentages of cesarean birth deliveries, gestational diabetes, gestational hypertension, preeclampsia, postpartum depression, and stillbirths compared to women without any clinical diagnoses of sleep disturbances. This trend persisted among women with one sleep disorder (*n* = 2701) and was more pronounced among women with two or more sleep disorders (*n* = 498), as shown in Table S2.

**TABLE 3:** Maternal outcomes by sleep disorders status of pregnant women enrolled in Clinformatics between January 1, 2015 and June 30, 2021.

Outcome	Total *N*	Sleep disorders (absent)	Sleep disorders (present)	*p* value
	93,645	90,446	3199	
Cesarean section				<0.0001
Yes	9426	8983 (9.93)	443 (13.85)	
No	84,219	81,463 (90.07)	2756 (86.15)	
Gestational diabetes				<0.0001
Yes	9197	8721 (9.64)	476 (14.88)	
No	84,448	81,725 (90.36)	2723 (85.12)	
Gestational hypertension				<0.0001
Yes	10,195	9627 (10.64)	568 (17.76)	
No	83,450	80,819 (89.36)	2631 (82.24)	
Preeclampsia				<0.0001
Yes	6917	6480 (7.16)	437 (13.66)	
No	86,728	83,966 (92.84)	2762 (86.34)	
Postpartum depression				<0.0001
Yes	2369	2120 (2.34)	249 (7.78)	
No	91,276	88,326 (97.66)	2950 (92.22)	
Stillbirth				<0.0001
Yes	502	460 (0.51)	42 (1.31)	
No	93,143	89,986 (99.49)	3157 (98.69)	

**FIGURE 3:**
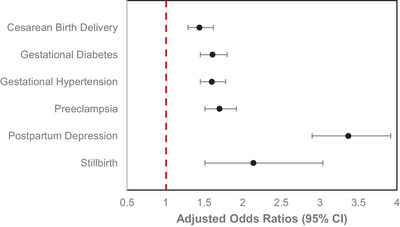
Adjusted odds ratios and 95% confidence intervals for the association between maternal sleep disorders and maternal outcomes. Models are adjusted for maternal age, maternal race, geographical division, and insurance‐related factors, including administrative services only, consumer‐driven health plan status, health exchange enrollment, and product type (*n* = 75,798). Each adjusted OR and 95% CI represents the odds of a documented maternal outcome diagnosis (yes) among women with any clinically diagnosed sleep disorder or breathing abnormality compared to those without. CI, confidence interval.

After adjusting for demographic factors and insurance‐related factors, these associations remained statistically significant (Figure 3). Maternal sleep disorders were independently associated with increased odds of cesarean birth delivery (aOR: 1.44; 95% CI: 1.29, 1.62), gestational diabetes (aOR: 1.61; 95% CI: 1.45, 1.80), gestational hypertension (aOR: 1.60; 95% CI: 1.45, 1.78), preeclampsia (aOR: 1.70; 95% CI: 1.51, 1.92), postpartum depression (aOR: 3.37; 95% CI: 2.90, 3.92), and stillbirth (aOR: 2.14; 95% CI: 1.51, 3.04). These positive associations strengthened with increasing sleep disorder burden: Individuals with two or more sleep disorders had significantly higher odds of each outcome compared to those with only one disorder (Table S3).

Table 4 presents the unadjusted associations between clinically diagnosed sleep disorders and breathing abnormalities with birth outcomes. Most women delivered a term newborn (87.17%), of AGA (96.21%), with normal birthweight (89.96%). Pregnant women with sleep disorders differed significantly from pregnant women without any sleep disorders in all birth outcomes examined. Although the majority of the women included in the study delivered newborns appropriate‐for‐gestational‐age, pregnant women with sleep disorders had higher percentages of delivering newborns SGA (4.16% vs. 3.06%) and LGA (1.01% vs. 0.69%). Birthweight distribution also varied between the groups even though the majority of the newborns were born with normal birthweight. There were higher percentages of newborns with LBW (6.68% vs. 3.64%) and HBW (7.50% vs. 6.26%) born to mothers with sleep disorders compared to mothers without sleep disorders. Although the majority of women in both groups delivered a term newborn, there were significant differences in their gestation period. Women with sleep disorders had a higher percentage of preterm births (13.61% vs. 7.37%) and a lower percentage of post‐term births (4.01% vs. 5.30%) compared to mothers without sleep disorders. A similar trend in the distribution of adverse birth outcomes was observed by the number of sleep disorders, as shown in Table S2. With the exception of post‐term births, pregnant women with one sleep disorder had higher prevalences of SGA, LGA, LBW, HBW, and preterm births compared to those without sleep disorders. This trend persisted for LGA, LBW, and preterm births among women with two or more sleep disorders.

**TABLE 4:** Birth outcomes by sleep disorders status of pregnant women enrolled in Clinformatics between January 1, 2015 and June 30, 2021.

Birth outcome	Total *N*	Sleep disorders (absent)	Sleep disorders (present)	*p* value
	76,656	74,082	2574	
Gestational age^a^				0.0010
AGA	73,745	71,304 (96.25)	2441 (94.83)	
SGA^b^	2371	2264 (3.06)	107 (4.16)	
LGA^c^	538	512 (0.69)	26 (1.01)	
Birthweight^d^				<0.0001
Normal	68,928	66,719 (90.10)	2209 (85.82)	
LBW^e^	2866	2694 (3.64)	172 (6.68)	
HBW^f^	4826	4633 (6.26)	193 (7.50)	
Gestation period^g^				<0.0001
Term	66,804	64,686 (87.33)	2118 (82.38)	
Preterm^h^	5809	5459 (7.37)	350 (13.61)	
Post‐term^i^	4026	3923 (5.30)	103 (4.01)	

^a^Missing (*n* = 2).

^b^Newborn whose weight and length are below the 10th percentile for gestational age.

^c^Newborn whose weight is more than 4500 g.

^d^Missing (*n* = 36).

^e^Newborn whose weight is less than 2500 g.

^f^Newborn whose weight is ≥4000 g.

^g^Missing (*n* = 17).

^h^Newborn with a gestation period of less than 37 completed weeks.

^i^Newborn with a gestation period over 40–42 completed weeks.

Abbreviations: AGA, appropriate gestational age; SGA, small for gestational age; LGA, large for gestational age; LBW, low birthweight; HBW, high birthweight.

**FIGURE 4:**
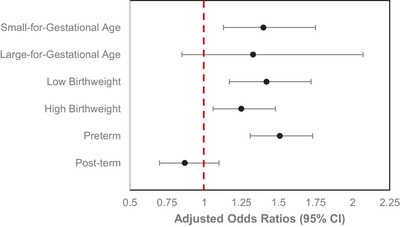
Adjusted odds ratios and 95% confidence intervals for the association between maternal sleep disorders and birth outcomes. Models are adjusted for maternal age, maternal race, infant's sex, geographical division, insurance‐related factors (administrative services only, consumer‐driven health plan, health exchange enrollment, and product type), and maternal pregnancy complications (gestational diabetes, gestational hypertension, preeclampsia, and mode of birth delivery). Sample sizes were newborn size by gestational age (*n* = 58,102), birthweight (*n* = 58,075), and gestational period (*n* = 58,094). Each adjusted OR and 95% CI represents the odds of a documented birth outcome diagnosis (yes) among women with any clinically diagnosed sleep disorder or breathing abnormality compared to those without. Reference groups for birth outcomes were mothers without any diagnosed sleep disorders who delivered infants that were appropriate for gestational age, had normal birthweight, and were born at term.

Overall, we found statistically significant associations between maternal sleep disorders and birth outcomes after adjusting for demographic factors, insurance‐related factors, infant's sex, and pregnancy complications (Figure 4). There was a significant 40% increase in odds of SGA and a suggestive but not significant 33% increase in odds of LGA compared to AGA among pregnant women with sleep disorders compared to those without any sleep disorders ([aOR: 1.40; 95% CI: 1.13, 1.75], [aOR: 1.33; 95% CI: 0.85, 2.07], *p* = 0.0047). For birthweight, both low and HBW had a significant 42% and 25% increase in odds, respectively, compared to normal birthweight among exposed pregnant women compared to those unexposed ([aOR: 1.42; 95% CI: 1.17, 1.72], [aOR: 1.25; 95% CI: 1.06, 1.48], *p* < 0.0001). Although there was an overall significant association with gestation period, the direction of the association differed for preterm and post‐term births. Compared to pregnant women without any sleep disorders, exposed pregnant women had a significant 51% increase in odds of preterm births and a 13% reduction in post‐term births compared to term births ([aOR: 1.51; 95% CI: 1.31, 1.73], [aOR: 0.87; 95% CI: 0.70, 1.10], *p* < 0.0001). Additionally, we observed a similar significant overall dose‐dependent relationship between the number of sleep disorders and birth outcomes; however, statistical significance was not reached across all exposure levels (e.g., two or more sleep disorders), as shown in Table S4.

## 4 Discussion

Our study examined the association between clinically diagnosed sleep disorders and breathing abnormalities and maternal and birth outcomes using objective measures based on clinical diagnoses. Over a period of 6.5 years, we found a 3.41% prevalence of clinically diagnosed sleep disorders and breathing abnormalities among women with singleton pregnancies. It was lower than previous observational studies using self‐reported measures (Amyx et al. 2017). The most prevalent sleep disorder was insomnia at 30.98%, which falls within the range reported in early pregnancy (12%–38%) but is lower than the estimates reported in mid‐pregnancy (59.7%), the third trimester (42.4%), and postpartum (55.2%) (Okun et al. 2015; Facco et al. 2010; Osnes et al. 2021; Salari et al. 2021). The second most prevalent sleep disorder was sleep apnea at 14.02%, with most diagnoses belonging to obstructive sleep apnea (OSA) (64.56%), which is considered the most common sleep‐related breathing disorder, ranging from 10.1% to 31.7% during pregnancy (Amyx et al. 2017; Maniaci et al. 2024; Pien et al. 2014). We included the diagnosis for snoring because it is considered a symptom of OSA with prevalence during pregnancy ranging from 14% to 45% during the third trimester, yet it only accounted for 3.65% of the diagnoses identified in our study sample (Silvestri and Arico 2019). Similarly, the prevalence of restless leg syndrome (3.98%) and parasomnia (0.20%) was far from their previous estimated ranges of 26%–30% and 5.7%–13.3%, respectively (Amyx et al. 2017; Silvestri and Arico 2019). This discrepancy between self‐reported and clinically diagnosed prevalence likely reflects underdiagnosis in healthcare settings. Several factors may contribute to this undercapture in administrative data, including limited provider screening, stigma around reporting sleep‐related concerns, and the absence of routine sleep assessments in prenatal care. Additionally, some patients may not seek care for milder symptoms, or providers may under‐document sleep complaints, particularly in busy obstetric settings where competing priorities limit time.

Using an objective approach to identify sleep disorders and maternal, fetal, and neonatal outcomes, we were able to examine their association among a large sample of pregnant women. We found significant demographic differences between pregnant women with and without sleep disorders and breathing abnormalities; these differences were consistent across most demographics except for health insurance‐related factors. Our results showed that sleep disorders, including breathing abnormalities, were associated with increased odds of adverse maternal outcomes (e.g., cesarean delivery, gestational diabetes, gestational hypertension, preeclampsia, postpartum depression, and stillbirth) and birth outcomes (e.g., newborn size by gestational age, birthweight, and gestation period). Our findings align with other studies that have examined these associations using self‐reported information (e.g., questionnaires) and a combination with objective measures (e.g., clinical data from medical and insurance claims records) (Balserak and Lee 2017; Moghadam et al. 2021; Akashanand et al. 2025; Takelle et al. 2022; Amyx et al. 2017; Steinweg et al. 2020; Wang et al. 2022; Yang et al. 2022; Lu et al. 2021; Li et al. 2018; Maghami et al. 2021; Brown et al. 2018; Warland et al. 2018; Maniaci et al. 2024; Komada et al. 2025). Although our study reflects associations rather than causation, the consistency of findings across multiple outcomes highlights the clinical importance of addressing sleep hygiene practices and implementing routine screening into prenatal care, which could be accomplished through brief, validated questionnaires administered during prenatal visits. Early identification and management of sleep disorders (e.g., continuous positive airway pressure [CPAP] for OSA) may improve maternal and neonatal outcomes and should be a focus of clinical guidelines (Nugent et al. 2023).

A major strength of our study was the utilization of available insurance health claims data, allowing the investigation of multiple outcomes in a large, nationally representative sample of women with singleton pregnancies. Additionally, standardized coding systems ensured consistency in sleep diagnoses confirmed by clinicians rather than self‐reported information, reducing recall or reporting bias. To minimize the overestimation of sleep diagnoses in our dataset, we included only pregnant women with continuous enrollment 1 year prior and 1 year after the culmination of their pregnancy, ensuring data completeness. This allowed us to determine a more accurate and reliable prevalence estimate of clinically diagnosed sleep disorders and breathing abnormalities, as well as the outcomes of interest.

Our study is not without limitations. We could not internally validate all of the captured sleep disorder diagnoses with sleep studies or polysomnography results or externally validate them with medical records. However, as our focus was on estimating the period prevalence, the presence of at least one claim record was deemed sufficient to indicate the sleep disorder diagnosis. We also lacked information on sociodemographic factors that may lead or confound the relationship between sleep disorders and maternal and birth outcomes; however, the insurance characteristics provide insights into the socioeconomic status and employment benefits, as different types of health insurance plans can be indicative of varying levels of employment benefits and financial status. We also lacked information on several potential confounders, such as BMI, parity, substance use, and comorbid mental health conditions, which may influence both sleep disorder risk and pregnancy outcomes (Facco et al. 2010; Osnes et al. 2021; Silvestri and Arico 2019). In addition, the use of ICD codes as proxies for clinical diagnosis carries the risk of misclassification, not only due to the potential for milder or subclinical presentations being underrepresented but also because of human error in entering diagnostic codes. Furthermore, our study population was restricted to women with commercial insurance, which limits the generalizability of our findings to the broader population of pregnant women in the United States. Sleep disorder prevalence and maternal and birth outcomes might differ in Medicaid‐insured or uninsured populations, who often face greater socioeconomic challenges and healthcare access barriers. Although we employed a one‐diagnosis‐per‐person approach to estimate the period prevalence of sleep disorders among pregnant women, future research should focus on prospective studies that evaluate the recurrence and severity of sleep disorders and breathing abnormalities to better understand their progression over time. Such studies could enhance diagnostic accuracy by combining administrative data with clinical chart reviews or objective sleep measurements (e.g., actigraphy or polysomnography). Including publicly insured or uninsured populations would also help to better understand disparities in diagnosis and care.

In conclusion, our findings align with previous research examining the relationship between sleep disorders and adverse maternal and birth outcomes. However, the prevalence of clinically diagnosed sleep disorders in our sample was substantially lower than estimates based on self‐report, highlighting a gap in detection and care. Given the observational nature of the data and limitations in clinical detail, caution is warranted when interpreting causal relationships. Nonetheless, the results underscore the need for better integration of sleep disorder screening into prenatal care and point to opportunities for future research to evaluate targeted interventions aimed at improving maternal and neonatal health outcomes through better sleep care.

## Author Contributions

**Anayeli Herrera Morales**: conceptualization, data curation, formal analysis, visualization, writing – original draft, methodology, investigation, writing – review and editing, software, validation. **Audrey C. Choh**: formal analysis, writing – review and editing, validation, methodology. **Cici X. Bauer**: methodology, writing – review and editing. **Stefan A. Czerwinski**: methodology, writing – review and editing. **Miryoung Lee**: conceptualization, data curation, formal analysis, methodology, supervision, project administration, writing – review and editing, resources.

## Conflicts of Interest

The authors declare no conflicts of interest.

## Peer Review

The peer review history for this article is available at https://publons.com/publon/10.1002/brb3.70908.

## Supporting information




**Supplementary Materials**: brb370908‐sup‐0001‐SuppMat.docx

## Data Availability

The extracted data that support the findings of this study cannot be made publicly available per our data use agreement.

## References

[brb370908-bib-0001] Ailes, E. C. , W. Zhu , E. A. Clark , et al. 2023. “Identification of Pregnancies and Their Outcomes in Healthcare Claims Data, 2008–2019: An Algorithm.” PLoS ONE 18, no. 4: e0284893. 37093890 10.1371/journal.pone.0284893 PMC10124843

[brb370908-bib-0002] Akashanand , P. Raghuveer , R. Yadav , and D. S. Reddy . 2025. “Prevalence and Determinants of Sleep Disturbances Among Pregnant Women: An Indian Community‐Based Cross‐Sectional Study.” Sleep and Biological Rhythms 23, no. 2: 127–136. 40190602 10.1007/s41105-024-00556-7 PMC11971108

[brb370908-bib-0003] American Medical Association . 2008. “International Classification of Diseases.” In Clinical Modification. 9th Revision. American Medical Association.

[brb370908-bib-0004] Amyx, M. , X. Xiong , Y. Xie , et al. 2017. “Racial/Ethnic Differences in Sleep Disorders and Reporting of Trouble Sleeping among Women of Childbearing Age in the United States.” Maternal and Child Health Journal 21, no. 2: 306–314. 27439422 10.1007/s10995-016-2115-9 PMC5250592

[brb370908-bib-0005] Armstrong, M. A. , D. A. Postlethwaite , J. A. Darbinian , et al. 2017. “Are Health Plan Design and Prior Use of Long‐Acting Reversible Contraception Associated With Pregnancy Intention?” Journal of Womens Health (2002) 26, no. 5: 450–460. 10.1089/jwh.2014.5146 27753522

[brb370908-bib-0041] American College of Obstetricians and Gynecologists . 2023. “Treatment and Management of Mental Health Conditions During Pregnancy and Postpartum: ACOG Clinical Practice Guideline No. 5.” Obstetrics and Gynecology 141, no. 6: 1262–1288. 37486661 10.1097/AOG.0000000000005202

[brb370908-bib-0006] Balserak, B. I. , and K. A Lee . 2017. “Sleep and Sleep Disorders Associated With Pregnancy.” In Principles and Practice of Sleep Medicine, edited by M. H. Kryger , and T. Roth , Elsevier. 1525–1539.

[brb370908-bib-0007] Brown, N. T. , J. M. Turner , and S Kumar . 2018. “The Intrapartum and Perinatal Risks of Sleep‐Disordered Breathing in Pregnancy: A Systematic Review and Meta‐Analysis.” American Journal of Obstetrics and Gynecology 219, no. 2: 147–161.e1. 29454869 10.1016/j.ajog.2018.02.004

[brb370908-bib-0008] Bureau of the Census, US Department of Commerce . n.d. *Census Bureau Regions and Division* With *State FIPS Codes* . Bureau of the Census, US Department of Commerce. https://www2.census.gov/geo/pdfs/maps‐data/maps/reference/us_regdiv.pdf.

[brb370908-bib-0009] Coelho, G. A 2022. “Sleep and Gender Differences.” In Sleep Medicine and Physical Therapy: A Comprehensive Guide for Practitioners, edited by C. Frange , and F. Coelho , Springer. 275–284.

[brb370908-bib-0010] de la Calle, M. , J. L. Bartha , and A. Martin Mens , et al. 2024. “Assessment of Sleep Quality in Spanish Twin Pregnancy: An Observational Single‐Center Study.” Twin Research and Human Genetics 27, no. 2: 97–104. 38505981 10.1017/thg.2024.13

[brb370908-bib-0011] Driver, H. S. , and E. P Sloan . 2017. “Sleep and Sleep Disorders in Women.” In Sleep Disorders Medicine: Basic Science, Technical Considerations, and Clinical Aspects, S. Chokroverty , Springer. 1159–1174.

[brb370908-bib-0012] Facco, F. L. , J. Kramer , K. H. Ho , et al. 2010. “Sleep Disturbances in Pregnancy.” Obstetrics and Gynecology 115, no. 1: 77–83. 20027038 10.1097/AOG.0b013e3181c4f8ec

[brb370908-bib-0013] American College of Obstetricians Gynecologists' Committee on Clinical Consensus‐Obstetrics . Gantt, A. , Society for Maternal‐Fetal Medicine . et al. 2022. “Obstetric Care Consensus #11, Pregnancy at Age 35 Years or Older.” American Journal of Obstetrics and Gynecology 140, no. 2: 348–366. 10.1097/AOG.0000000000004873 35852294

[brb370908-bib-0014] Gavrielov‐Yusim, N. , and M Friger . 2014. “Use of Administrative Medical Databases in Population‐Based Research.” Journal of Epidemiology and Community Health 68, no. 3: 283–287. 24248997 10.1136/jech-2013-202744

[brb370908-bib-0015] Hauspurg, A. , and A Jeyabalan . 2022. “Postpartum Preeclampsia or Eclampsia: Defining Its Place and Management Among the Hypertensive Disorders of Pregnancy.” American Journal of Obstetrics and Gynecology 226, no. 2S: S1211–S1221. 35177218 10.1016/j.ajog.2020.10.027 PMC8857508

[brb370908-bib-0016] Jolley, R. J. , Z. Liang , M. Peng , et al. 2018. “Identifying Cases of Sleep Disorders Through International Classification of Diseases (ICD) Codes in Administrative Data.” International Journal of Population Data Science 3, no. 1: 448. 32935008 10.23889/ijpds.v3i1.448 PMC7299484

[brb370908-bib-0017] Komada, Y. , S. I. Kawakami , S. Furuie , et al. 2025. “Association Between Sleep Problems During Pregnancy and Postpartum Depressive Symptoms as Well as Condition of Newborn at Delivery.” Journal of Obstetrics and Gynaecology Research 51, no. 2: e16219. 39865440 10.1111/jog.16219 PMC11771626

[brb370908-bib-0018] Laopaiboon, M. , P. Lumbiganon , N. Intarut , et al. 2014. “Advanced Maternal Age and Pregnancy Outcomes: A Multicountry Assessment.” BJOG 121, no. S1: 49–56. 24641535 10.1111/1471-0528.12659

[brb370908-bib-0019] Lee, D. C. , H. Liang , and L Shi . 2021. “The Convergence of Racial and Income Disparities in Health Insurance Coverage in the United States.” International Journal for Equity in Health 20, no. 1: 96. 33827600 10.1186/s12939-021-01436-z PMC8025443

[brb370908-bib-0020] Li, L. , K. Zhao , J. Hua , et al. 2018. “Association Between Sleep‐Disordered Breathing During Pregnancy and Maternal and Fetal Outcomes: An Updated Systematic Review and Meta‐Analysis.” Frontiers in Neurology 9: 91. 29892255 10.3389/fneur.2018.00091 PMC5985400

[brb370908-bib-0021] Lu, Q. , X. Zhang , Y. Wang , et al. 2021. “Sleep Disturbances During Pregnancy and Adverse Maternal and Fetal Outcomes: A Systematic Review and Meta‐Analysis.” Sleep Medicine Reviews 58: 101436. 33571887 10.1016/j.smrv.2021.101436

[brb370908-bib-0022] Pavlova, M. K. , and V. Latreille . 2019. “Sleep Disorders.” American Journal of Medicine 132, no. 3: 292–299. 30292731 10.1016/j.amjmed.2018.09.021

[brb370908-bib-0023] Maghami, M. , S. P. Shariatpanahi , D. Habibi , et al. 2021. “Sleep Disorders During Pregnancy and Postpartum Depression: A Systematic Review and Meta‐Analysis.” International Journal of Developmental Neuroscience 81, no. 6: 469–478. 33942364 10.1002/jdn.10118

[brb370908-bib-0024] Maniaci, A. , L. La Via , and B. Pecorino , et al. 2024. “Obstructive Sleep Apnea in Pregnancy: A Comprehensive Review of Maternal and Fetal Implications.” Neurology International 16, no. 3: 522–532. 38804478 10.3390/neurolint16030039 PMC11130811

[brb370908-bib-0025] Moghadam, Z. B. , E. Rezaei , and A Rahmani . 2021. “Sleep Disorders during Pregnancy and Postpartum: A Systematic Review.” Sleep Medicine Research 12, no. 2: 81–93.

[brb370908-bib-0026] Nugent, R. , A. Wee , L. Kearney , et al. 2023. “The Effectiveness of Continuous Positive Airway Pressure for Treating Obstructive Sleep Apnoea in Pregnancy: A Systematic Review.” Australian and New Zealand Journal of Obstetrics and Gynaecology 63, no. 3: 290–300. 36866618 10.1111/ajo.13654

[brb370908-bib-0027] Okun, M. L. , D. J. Buysse , and M. H Hall . 2015. “Identifying Insomnia in Early Pregnancy: Validation of the Insomnia Symptoms Questionnaire (ISQ) in Pregnant Women.” Journal of Clinical Sleep Medicine 11, no. 6: 645–654. 25766716 10.5664/jcsm.4776 PMC4442225

[brb370908-bib-0028] Osnes, R. S. , M. Eberhard‐Gran , T. Follestad , et al. 2021. “Mid‐Pregnancy Insomnia and Its Association With Perinatal Depressive Symptoms: A Prospective Cohort Study.” Behavioral Sleep Medicine 19, no. 3: 285–302. 32228307 10.1080/15402002.2020.1743705

[brb370908-bib-0029] Osterman, M. , B. Hamilton , J. A. Martin , et al. 2021. “Births: Final Data for 2020.” National Vital Statistics Reports 70, no. 17: 1–50. 35157571

[brb370908-bib-0030] Pien, G. W. , A. I. Pack , N. Jackson , et al. 2014. “Risk Factors for Sleep‐Disordered Breathing in Pregnancy.” Thorax 69, no. 4: 371–377. 24262432 10.1136/thoraxjnl-2012-202718 PMC6994201

[brb370908-bib-0031] Putnick, D. L. , R. Sundaram , E. M. Bell , et al. 2020. “Trajectories of Maternal Postpartum Depressive Symptoms.” Pediatrics 146, no. 5: e20200857. 33109744 10.1542/peds.2020-0857 PMC7772818

[brb370908-bib-0032] Salari, N. , N. Darvishi , B. Khaledi‐Paveh , et al. 2021. “A Systematic Review and Meta‐Analysis of Prevalence of Insomnia in the Third Trimester of Pregnancy.” BMC Pregnancy Childbirth 21, no. 1: 284. 33836686 10.1186/s12884-021-03755-z PMC8034118

[brb370908-bib-0033] Santana, D. S. , C. Silveira , M. L. Costa , et al. 2018. “Perinatal Outcomes in Twin Pregnancies Complicated by Maternal Morbidity: Evidence From the WHO Multicountry Survey on Maternal and Newborn Health.” BMC Pregnancy Childbirth 18, no. 1: 449. 30453908 10.1186/s12884-018-2082-9 PMC6245698

[brb370908-bib-0034] SAS Institute Inc . 2023. SAS/STAT Software, Version 9.4. SAS Institute Inc.. https://www.sas.com.

[brb370908-bib-0035] Silvestri, R. , and I Arico . 2019. “Sleep Disorders in Pregnancy.” Sleep Science 12, no. 3: 232–239. 31890101 10.5935/1984-0063.20190098 PMC6932848

[brb370908-bib-0036] Steinweg, K. , T. Nippita , P. A. Cistulli , et al. 2020. “Maternal and Neonatal Outcomes Associated With Restless Legs Syndrome in Pregnancy: A Systematic Review.” Sleep Medicine Reviews 54: 101359. 32805557 10.1016/j.smrv.2020.101359

[brb370908-bib-0037] Takelle, G. M. , N. Y. Muluneh , and M. S Biresaw . 2022. “Sleep Quality and Associated Factors Among Pregnant Women Attending Antenatal Care Unit at Gondar, Ethiopia: A Cross‐Sectional Study.” BMJ Open 12, no. 9: e056564. 10.1136/bmjopen-2021-056564 PMC9445783 36691143

[brb370908-bib-0038] The Association of Health Care Journalists . n.d. Insurance Glossary. Association of Health Care Journalists. https://healthjournalism.org/glossary/insurance‐glossary/.

[brb370908-bib-0039] The Centers for Medicare and Medicaid Services . n.d. U.S. Department of Health and Human Services . HealthCare.gov. https://www.healthcare.gov/glossary/.

[brb370908-bib-0040] The National Center for Health Statistics . n.d. “US Department of Health and Human Services.” In International Classification of Diseases, Tenth Revision, Clinical Modification (ICD‐10‐CM) Browser Tool. National Center for Health Statistics. https://icd10cmtool.cdc.gov/.

[brb370908-bib-0042] Wang, R. , M. Xu , W. Yang , et al. 2022. “Maternal Sleep During Pregnancy and Adverse Pregnancy Outcomes: A Systematic Review and Meta‐Analysis.” Journal of Diabetes Investigation 13: 1262–1276. 35171528 10.1111/jdi.13770 PMC9248434

[brb370908-bib-0043] Warland, J. , J. Dorrian , J. L. Morrison , et al. 2018. “Maternal Sleep During Pregnancy and Poor Fetal Outcomes: A Scoping Review of the Literature With Meta‐Analysis.” Sleep Medicine Reviews 41: 197–219. 29910107 10.1016/j.smrv.2018.03.004

[brb370908-bib-0044] World Health Organization . 2016. International Statistical Classification of Diseases and Related Health Problems. 10th Revision. World Health Organization.

[brb370908-bib-0045] Yang, Z. , Z. Zhu , C. Wang , et al. 2022. “Association Between Adverse Perinatal Outcomes and Sleep Disturbances During Pregnancy: A Systematic Review and Meta‐Analysis.” Journal of Maternal‐Fetal & Neonatal Medicine 35, no. 1: 166–174. 31902261 10.1080/14767058.2020.1711727

