# Supplementary material for: Maternal Sleep Disorders and Maternal and Birth Outcomes: A Retrospective US Claims‐Based Study
Source: Brain Behav. 2025 Sep 25;15(9):e70908. doi: 10.1002/brb3.70908 (PMC12463701; doi:10.1002/brb3.70908)
Supplement: Supplementary file 1 — Supplementary Materials: brb370908‐sup‐0001‐SuppMat.docx [file BRB3-15-e70908-s001.docx]

**Supporting Information**

| Table S1. Distribution of sleep disorders by ICD-10 categories and subcategories for pregnant women enrolled in Clinformatics^®^ from January 1, 2015, to June 30, 2021. | |
| --- | --- |
| Sleep and breathing abnormalities disorder | n = 3,944^a^ |
| Sleep disorders not due to a substance or known physiological condition | |
| *F51.0 insomnia* | |
| F51.01 – primary insomnia | 123 |
| F51.02 – adjustment insomnia | 38 |
| F51.04 – psychophysiologic insomnia | 68 |
| F51.05 – insomnia due to other mental disorders | 42 |
| F51.09 – other insomnia not due to a substance or known physiological condition | 15 |
| *F51.1 hypersomnia* | |
| F51.11 – primary hypersomnia | N<5^b^ |
| F51.12 – insufficient sleep syndrome | N<5^b^ |
| F51.13 – hypersomnia due to other mental disorder | N<5^b^ |
| F51.19 – other hypersomnia not due to a substance or known physiological condition | N<5^b^ |
| *F51 other sleep disorders not due to a substance or known physiological condition* | |
| F51.3 – sleepwalking somnambulism | 5 |
| F51.4 – sleep terrors night terrors | 5 |
| F51.5 – nightmare disorder | 13 |
| F51.8 – other sleep disorders not due to a substance or known physiological condition | N<5 |
| F51.9 – sleep disorder not due to a substance or known physiological condition, unspecified | 6 |
| Other extrapyramidal and movement disorders | |
| *G25.8 other specified extrapyramidal and movement disorders* | |
| G25.81 – restless legs syndrome | 157 |
| Sleep disorders | |
| *G47.0 insomnia* | |
| G47.00 – insomnia, unspecified | 846 |
| G47.01 – insomnia due to medical condition | 27 |
| G47.09 – other insomnia | 63 |
| *G47.1 hypersomnia* | |
| G47.10 – hypersomnia, unspecified | 74 |
| G47.11 – idiopathic hypersomnia with long sleep time | 8 |
| G47.12 – idiopathic hypersomnia without long sleep time | N<5^b^ |
| G47.13 – recurrent hypersomnia | N<5^b^ |
| G47.14 – hypersomnia due to medical condition | N<5^b^ |
| G47.19 – other hypersomnia | 27 |
| *G47.2 circadian rhythm sleep disorders* | |
| G47.20 – circadian rhythm sleep disorder, unspecified type | 11 |
| G47.21 – circadian rhythm sleep disorder, delayed sleep phase type | 5 |
| G47.23 – circadian rhythm sleep disorder, irregular sleep wake type | 13 |
| G47.26 – circadian rhythm sleep disorder, shift work type | 8 |
| *G47.3 sleep apnea* | |
| G47.30 – sleep apnea, unspecified | 179 |
| G47.31 – primary central sleep apnea | N<5^b^ |
| G47.33 – obstructive sleep apnea (adult) (pediatric) | 357 |
| G47.34 – idiopathic sleep-related nonobstructive alveolar hypoventilation | N<5^b^ |
| G47.36 – sleep-related hypoventilation in conditions classified elsewhere | N<5^b^ |
| G47.39 – other sleep apnea | 9 |
| *G47.4 narcolepsy and cataplexy* | |
| G47.411 – narcolepsy with cataplexy | 14 |
| G47.419 – narcolepsy without cataplexy | 38 |
| G47.429 – narcolepsy in conditions classified elsewhere without cataplexy | 5 |
| *G47.5 parasomnia* | |
| G47.50 – parasomnia, unspecified | N<5^b^ |
| G47.51 – confusional arousals | N<5^b^ |
| G47.52 – rem sleep behavior disorder | N<5^b^ |
| G47.53 – recurrent isolated sleep paralysis | N<5^b^ |
| *G47.6 sleep-related movement disorders* | |
| G47.61 – periodic limb movement disorder | 8 |
| G47.62 – Sleep-related leg cramps | 10 |
| G47.63 – sleep related bruxism | 11 |
| *G47 other or unspecified sleep disorders* | |
| G47.8 – other sleep disorders | 30 |
| G47.9 – sleep disorder, unspecified | 136 |
| Abnormalities of breathing | |
| *R06 abnormalities of breathing* | |
| R06.00 – dyspnea, unspecified | 1,082 |
| R06.01 – orthopnea | 30 |
| R06.09 – other forms of dyspnea | 265 |
| R06.1 – stridor | 13 |
| R06.3 – periodic breathing | N<5^b^ |
| R06.81 – apnea, not elsewhere classified | 22 |
| R06.83 – snoring | 144 |
| ^a^: As sleep disorders are not mutually exclusive, some women were diagnosed with more than one disorder during the study period.  ^b^: In accordance with our data use agreement, we do not report exact diagnosis counts for cells containing fewer than 5 individuals. | |

| Table S2. Maternal and birth outcomes by sleep disorders status of pregnant women enrolled in Clinformatics® between January 1, 2015, and June 30, 2021. | | | | | | | | |
| --- | --- | --- | --- | --- | --- | --- | --- | --- |
|  |  | **Number of sleep disorders** | | | | |  |  |
| Outcome | Total N | Zero | One | | Two or more | |  |  |
| Maternal | 93,645 | 90,446 | | 2,701 | | 498 | |  |
| Cesarean section |  |  | |  | |  | |  |
| Yes | 9,426 | 8,983 (9.93) | | 363 (13.44) | | 80 (16.06) | |  |
| No | 84,219 | 81,463 (90.07) | | 2,338 (86.56) | | 418 (83.94) | |  |
| Gestational diabetes |  |  | |  | |  | |  |
| Yes | 9,197 | 8,721 (9.64) | | 381 (14.11) | | 95 (19.08) | |  |
| No | 84,448 | 81,725 (90.36) | | 2,320 (85.89) | | 403 (80.92) | |  |
| Gestational hypertension |  |  | |  | |  | |  |
| Yes | 10,195 | 9,627 (10.64) | | 465 (17.22) | | 103 (20.68) | |  |
| No | 83,450 | 80,819 (89.36) | | 2,236 (82.78) | | 395 (79.32) | |  |
| Preeclampsia |  |  | |  | |  | |  |
| Yes | 6,917 | 6,480 (7.16) | | 334 (12.37) | | 103 (20.68) | |  |
| No | 86,728 | 83,966 (92.84) | | 2,367 (87.63) | | 395 (79.32) | |  |
| Postpartum depression |  |  | |  | |  | |  |
| Yes | 2,369 | 2,120 (2.34) | | 213 (7.89) | | 36 (7.23) | |  |
| No | 91,276 | 88,326 (97.66) | | 2,488 (92.11) | | 462 (92.77) | |  |
| Stillbirth |  |  | |  | |  | |  |
| Yes | 502 | 460 (0.51) | | 33 (1.22) | | 9 (1.81) | |  |
| No | 93,143 | 89,986 (99.49) | | 2,668 (98.78) | | 489 (98.19) | |  |
| Birth | 76,656 | 74,082 | | 2,178 | | 396 | |  |
| Gestational age ^a^ |  |  | |  | |  | |  |
| Appropriate | 73,745 | 71,304 (96.25) | | 2,066 (94.86) | | 375 (94.70) | |  |
| Small^†^ | 2,371 | 2,264 (3.06) | | 91 (4.18) | | 16 (4.04) | |  |
| Large^‡^ | 538 | 512 (0.69) | | 21 (0.96) | | 5 (1.26) | |  |
| Birthweight ^b^ |  |  | |  | |  | |  |
| Normal | 68,928 | 66,719 (90.10) | | 1,872 (85.95) | | 337 (85.10) | |  |
| Low ^+^ | 2,866 | 2,694 (3.64) | | 143 (6.57) | | 29 (7.32) | |  |
| High ^++^ | 4,826 | 4,633 (6.26) | | 163 (7.48) | | 30 (7.58) | |  |
| Gestation period ^c^ |  |  | |  | |  | |  |
| Term | 66,804 | 64,686 (87.33) | | 1,813 (83.28) | | 305 (77.41) | |  |
| Preterm ^ӿ^ | 5,809 | 5,459 (7.37) | | 279 (12.82) | | 71 (18.02) | |  |
| Post-term ^Ӿ^ | 4,026 | 3,923 (5.30) | | 85 (3.90) | | 18 (4.57) | |  |
| ^a^: Missing (n=2)  ^†^: Small for gestational age: newborn whose weight and length are below the 10^th^ percentile for gestational age  ^‡^: Large for gestational age: newborn whose weight is more than 4,500 grams  ^b^: Missing (n=36)  ^+^: Low birthweight: newborn whose weight is less than 2,500 grams  ^++^: High birthweight: newborn whose weight is ≥ 4,000 grams  ^c^: Missing (n=17)  ^ӿ^: Preterm: newborn with a gestation period of less than 37 completed weeks  ^Ӿ^: Post-term: newborn with a gestation period over 40 completed weeks to 42 completed weeks | | | | | | | | |

| Table S3. Associations* between the number of maternal sleep disorders and maternal outcomes. | | | | | | |
| --- | --- | --- | --- | --- | --- | --- |
|  | **Odds Ratio (95% Confidence Interval)** | | | | | |
|  | Cesarean birth delivery ^a^ | Gestational diabetes ^b^ | Gestational hypertension ^c^ | Preeclampsia ^d^ | Postpartum depression ^e^ | Stillbirth ^f^ |
| Sleep disorders |  |  |  |  |  |  |
| Zero | Reference | Reference | Reference | Reference | Reference | Reference |
| One | 1.40 (1.24, 1.59) | 1.55 (1.38, 1.75) | 1.55 (1.38, 1.74) | 1.53 (1.34,1.76) | 3.44 (2.92, 4.04) | 2.13 (1.45, 3.14) |
| Two or more | 1.67 (1.29, 2.17) | 1.94 (1.51, 2.49) | 1.88 (1.48, 2.39) | 2.62 (2.03, 3.37) | 3.06 (2.11, 4.43) | 2.17 (1.00, 4.67) |
| Overall significance | p<0.0001 | p<0.0001 | p<0.0001 | p<0.0001 | p<0.0001 | p=0.0001 |
| *: Models are adjusted for maternal age, maternal race, gestational age, geographical division, and factors related to health insurance (administrative services only, consumer-driven health plan, health exchange, and product); n=75,798 | | | | | | |

| Table S4. Associations* between the number of maternal sleep disorders^#^ and birth outcomes. | | | |
| --- | --- | --- | --- |
|  | Odds Ratio (95% Confidence Interval) | | |
| Sleep Disorders | Gestational Age ^a^ | Birthweight ^b^ | Gestation Period ^c^ |
| Zero | Reference | Reference | Reference |
|  | Appropriate for Gestational Age | Normal birthweight | Term |
|  | Small for Gestational Age^†^ | Low birthweight ^+^ | Preterm ^ӿ^ |
| 1 disorder | 1.45 (1.15, 1.84) | 1.42 (1.15, 1.76) | 1.41 (1.21, 1.65) |
| 2 or more | 1.17 (0.68, 2.03) | 1.41 (0.92, 2.17) | 2.00 (1.48, 2.70) |
|  | Large for Gestational Age^‡^ | High birthweight ^++^ | Post-term ^Ӿ^ |
| 1 disorder | 1.21 (0.74, 2.02) | 1.27 (1.06, 1.52) | 0.84 (0.65, 1.07) |
| 2 or more | 1.89 (0.78, 4.70) | 1.16 (0.77, 1.77) | 1.08 (0.64, 1.83) |
| Overall significance | p=0.0149 | p=0.0009 | p<0.0001 |
| *: Models are adjusted for maternal age, maternal race, infant’s sex, geographical division, insurance-related factors (administrative services only, consumer-driven health plan, health exchange, and product), and maternal pregnancy complications (gestational diabetes, gestational hypertension, preeclampsia, and mode of birth delivery)  ^#^: Zero sleep disorders as the reference group  ^a^: Appropriate for gestational age as the reference group; n=58,102  ^†^ Small for gestational age: newborn whose weight and length are below the 10^th^ percentile for gestational age  ^‡^ Large for gestational age: newborn whose weight is more than 4,500 grams  ^b^: Normal birthweight as the reference group; n=58,075  ^+^: Low birthweight: newborn whose weight is less than 2,500 grams  ^++^: High birthweight: newborn whose weight is ≥ 4,000 grams  ^c^: Term gestation period as the reference group; n=58,094  ^ӿ^: Preterm: newborn with a gestation period of less than 37 completed weeks  ^Ӿ^: Post-term: newborn with a gestation period over 40 completed weeks to 42 completed weeks | | | |
